# Systematic optimization of visible light-induced crosslinking conditions of gelatin methacryloyl (GelMA)

**DOI:** 10.1038/s41598-021-02830-x

**Published:** 2021-12-02

**Authors:** Sina Sharifi, Hannah Sharifi, Ali Akbari, James Chodosh

**Affiliations:** 1 Disruptive Technology Laboratory, Department of Ophthalmology, Harvard Medical School, Massachusetts Eye and Ear and Schepens Eye Research Institute, Boston, MA USA; 2 Solid Tumor Research Center, Research Institute for Cellular and Molecular Medicine, Urmia University of Medical Sciences, Urmia, Iran

**Keywords:** Biomaterials, Biomedical engineering

## Abstract

Gelatin methacryloyl (GelMA) is one of the most widely used photo-crosslinkable biopolymers in tissue engineering. In in presence of an appropriate photoinitiator, the light activation triggers the crosslinking process, which provides shape fidelity and stability at physiological temperature. Although ultraviolet (UV) has been extensively explored for photo-crosslinking, its application has been linked to numerous biosafety concerns, originated from UV phototoxicity. Eosin Y, in combination with TEOA and VC, is a biosafe photoinitiation system that can be activated via visible light instead of UV and bypasses those biosafety concerns; however, the crosslinking system needs fine-tuning and optimization. In order to systematically optimize the crosslinking conditions, we herein independently varied the concentrations of Eosin Y [(EY)], triethanolamine (TEOA), vinyl caprolactam (VC), GelMA precursor, and crosslinking times and assessed the effect of those parameters on the properties the hydrogel. Our data showed that except EY, which exhibited an optimal concentration (~ 0.05 mM), increasing [TEOA], [VA], [GelMA], or crosslinking time improved mechanical (tensile strength/modulus and compressive modulus), adhesion (lap shear strength), swelling, biodegradation properties of the hydrogel. However, increasing the concentrations of crosslinking reagents ([TEOA], [VA], [GelMA]) reduced cell viability in 3-dimensional (3D) cell culture. This study enabled us to optimize the crosslinking conditions to improve the properties of the GelMA hydrogel and to generate a library of hydrogels with defined properties essential for different biomedical applications.

## Introduction

Protein-based hydrogels including collagen, fibrin, elastin, silk, and gelatin have been extensively used in various biomedical applications. These materials are generally biocompatible, and because of their innate characteristics, can modulate cellular adhesion, proliferation, migration, and differentiation^1–4^. Gelatin methacryloyl (GelMA) is one of the most studied and versatile biopolymers to form a hydrogel used in biomedical engineering studies, such as micropatterning, fluidic systems, 3-D scaffolds, bioprinting with different cell types, tissue adhesives, and drug delivery^3,5^. GelMA mimics many important properties of native extracellular matrix (ECM), contains cell binding (e.g. Arg-Gly-Asp (RGD)) and matrix metalloproteinase (MMP)-sensitive degradation sites, and is biocompatible and tunable^6–8^. Taking into account the burgeoning interest in biopolymer-based hydrogels and the potential of GelMA, optimizing the efficacy of the preparation process^5,9^ and crosslinking are important.

To form a covalently crosslinked hydrogel network, the GelMA precursor undergoes a radical polymerization in the presence of a photoinitiator and light with an appropriate wavelength and intensity^5^. Ultraviolet (UV) is the most commonly used light to photo-crosslink GelMA and similar methacrylated materials such as tropoelastin^10^, elastin-like polypeptides^11^, poly(ethylene glycol) (PEG)-diacrylate hydrogels^12^, and many other elastomers^13,14^. However, there are several biosafety concerns associated with the application of UV light when it comes to using living cells and tissues. It was shown that UV light can cause damage either through pyrimidine dimerization or generation of reactive oxygen species (ROS) that may induce oxidative damage to DNA and lead to accelerated tissue aging, immunosuppression, and ultimately to cancer^15–17^. The combination of UV light and common UV photoinitiator (Irgacure 2959) also was shown to have harmful effects on cell viability, correlated with the irradiation time^18,19^. Besides such phototoxic effects, UV light has limited penetration to tissues and hydrogels, which adds an additional hurdle to its biomedical applications. These challenges have propelled the scientific community to explore the application of light with higher wavelengths including visible light in conjunction with a photoinitiator that can be activated in those wavelengths^20–22^. Various visible-light photoinitiation systems, including lithium phenyl-2,4,6-trimethylbenzoylphosphinate (LAP) (absorption peak of 405 nm), riboflavin (absorption peak of 440 and 371 nm), carboxylated camphorquinone, (absorption peak of 470 nm), and Eosin Y (EY) (absorption peak of 510 nm) have been explored in biomedical applications^23^. The initiation systems that use higher wavelengths (e.g. green light) for activation are generally safer than those with lower wavelength energy, as the energy of photons used for crosslinking has a direct correlation with its phototoxicity^24^. For example, LAP is a single-component initiation system with high thermal stability and good solubility and is colorless; however, it is activated by blue visible light, which was shown to have some level of toxicity^25^. Eosin Y (EY) system, on the other hand, is a two-component initiation system, in which EY acts as photosensitizer and triethanolamine (TEOA) acts as an initiator and is activated by visible light with higher wavelengths to generate free radicals for the polymerization reaction^26–28^. The resultant hydrogel is optically transparent and has a pale yellow color, which originates from the yellow color of the activated EY. To accelerate gelation kinetics, different co-monomers such as N-Vinylcaprolactam (VC) or 1-vinyl-2 pyrrolidinone (NVP) are usually added to the crosslinking solution^4,29^. EY system is also Food and Drug Administration (FDA)-approved for the application of photo-crosslinkable lung sealant FocalSeal® (Genzyme Biosurgical, Cambridge, MA). The EY system can bypass phototoxicity induced by UV light, but the chemical reagents used in the crosslinking system (EY, TEOA, and VC) are toxic in high concentrations—especially for cellular photoencapsulation, which has a wide range of applications including 3-D bioprinting, differentiation studies, drug discovery and pharmacological applications, cancer research, gene, and protein expression studies^30–33^. To understand how altering the crosslinking conditions impact properties of the hydrogel, we herein performed a systematic study in which we independently varied the concentration of crosslinking reagents (EY, TEOA, and VC), the concentration of GelMA precursor, and the crosslinking time and studied the resulting changes in chemical, physical, mechanical, and biological properties of the hydrogel.

## Results and discussion

### Tuning Eosin Y concentration

EY is a photosensitizer, used in combination with the TEOA (initiator) and VC (co-monomer) for photo-induced crosslinking reaction under visible light^28^. When the EY absorption overlaps with the irradiated light spectrum, EY absorbs the light and moves to an excited state. Excited EY then abstracts a hydrogen radical (H˙) from TEOA and generates a TEOA radical, which serves as an initiator of crosslinking^26–28,34^. Visible light-induced crosslinking often necessitates the addition of co-monomers, such as N-Vinylcaprolactam (VC) or 1-vinyl-2 pyrrolidinone (NVP) to accelerate gelation kinetics. The addition of a co-monomer has several impacts: (1) it increases the vinyl group concentration in the reaction media to synergistically cross‐propagate with methacrylate groups of GelMA; (2) it expedites a diffusion of radicalized compounds in the reaction media^35^; and (3) it facilitates free-radical polymerization through prevention of the scavenging the free-radicals by available oxygen^12,36^. These enhance the crosslinking density of the polymeric network and subsequently improve the structural and mechanical properties of the hydrogel^37^. Therefore, the crosslinking conditions are a valuable tool to control the physiochemical and biological characteristics of the hydrogel^26,35–41^. While extensive studies have been performed to understand the effects of polymerization conditions on the properties of polyethylene glycol (PEG)-based hydrogels^39,40,42,43^, limited reports have focused on the effects of polymerization conditions of the GelMA hydrogel. Noshadi et al*.* varied [TEOA] from 0.5 to 1.5% (v/v) and [VC] from 0.5 to 1.5% (w/v) while retaining [EY] at 0.1 mM, and studied the mechanical properties of crosslinked hydrogels under the visible light irradiation^26^. They found that the compressive and tensile moduli of the GelMA hydrogels (10% w/v) were directly correlated with [VC] and [TEOA], varying from 5–56.5 kPa, and 5 to 22.7 kPa, respectively. Recently, a modification in the crosslinking condition ( [TEOA] = 1.875% (w/v), [VC] = 1.25% (w/v), and [EY] = 0.5 mM) was shown to increase the elastic modulus and tensile strength up to 224.4 ± 32.3 and 45.3 ± 4.1 kPa, respectively when [GelMA precursor] is 20% (w/v) after 4 min irradiation with visible light (450–550 nm, and the intensity of 100 mW/cm^2^) ^22^.

To better understand how crosslinking conditions impact hydrogel properties, we herein performed a comprehensive systematic study and varied the concentration of crosslinking reagents in a wider range ([EY] = 0.005–1 mM; [TEOA] = 0.05–5% (w/v); [VC] = 0.05–5% (w/v); [GelMA precursor] = 5–20%); and time = 0.5 = 10 min) (Table S1) than in prior work.

**Figure 1:**
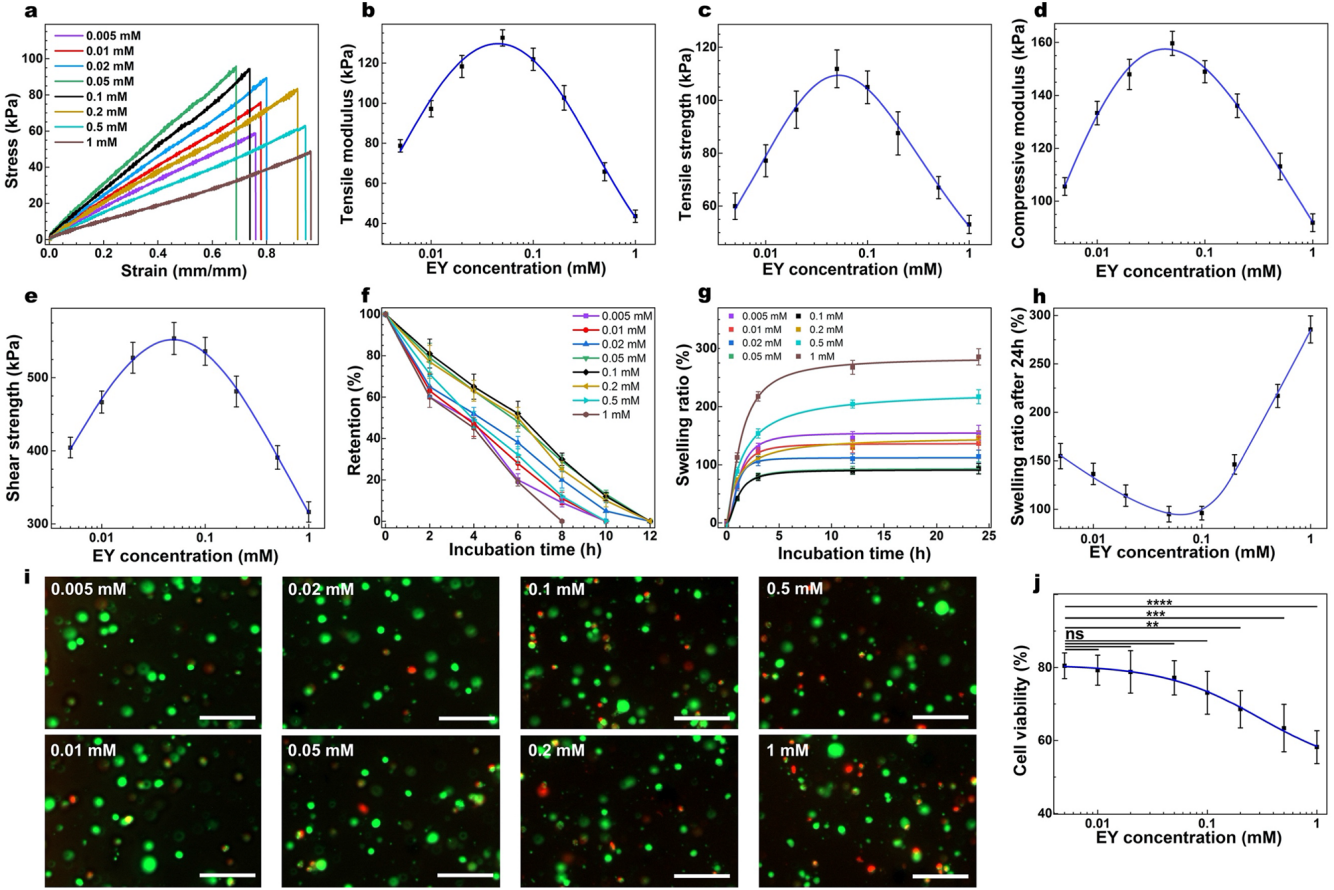
Tuning [EY] and its impact on the structural and biological properties of GelMA. **a**) Representative tensile stress/strain plots for GelMA (20% w/v and crosslinked for 1 min) with varying [EY] and corresponding average tensile modulus (**b**) and tensile strength (**c**), along with mean compressive modulus (**d**), and shear strength (**e**) as a function of [EY]. Collagenase induced degradation (**f**) and swelling ratio (**g**) of the GelMA hydrogels formed with varying [EY] (20% w/v) as a function of time, and corresponding swelling ratios (**h**) after 24 h of incubation at 37 °C. (**i**) Representative Live-Dead images of the encapsulated corneal fibroblasts (CF) in 3-dimensional (3-D) cell culture and the quantification of cell viability (**j**) after 1 day of post-seeding on GelMA hydrogels (10% w/v and crosslinked for 1 min) formed with varying [EY] (scale bars: 150 μm). Values are presented as mean ± SD; n = 4. ns, *, **, ***, and **** represent *p* > 0.05, *p* ≤ 0.05, *p* ≤ 0.01, *p* ≤ 0.001 and *p* ≤ 0.0001, respectively.

Successful polymerization reaction necessitates the generation of radical species with optimal concentrations. Such optimal concentrations assure that the reaction is not slow ([radical species] is low), or the reaction mixture does not contain too many free radicals in a close proximity, which can lead to smaller molecular weight chains ([radical species] is high). To generate those radical species, both EY and TEOA are necessary. Lower [EY] in the reaction leads to lower [activated EY], and lower [TEOA], and consequently fewer radical species, and having higher [EY] results in the opposite. Therefore, [radical species] can be approximately correlated with [EY]. However, at given [EY], if [TEOA] is too low, then those activated EY species are unable to activate TEOA to generate radicals, and if [TEOA] is too high, then the extra TEOA will remain unused. These indicate that the impact of EY is primary to that of TEOA. On that basis, we first tuned [EY] and then [TEOA]. Inspired by previous studies^5,22,26,28^, we selected [EY] from 0.005 to 1 mM (0.005, 0.01, 0.02, 0.05, 0.1, 0.2, 0.5, 1 mM), while maintaining the other parameters constant ( [TEOA] = 1% (w/v); [VC] = 0.5% (w/v); [GelMA precursor] = 20% (w/v); and time = 1 min). Our studies show that the tensile strength and elastic modulus of GelMA are strongly dependent on [EY] (Fig. 1a–c,). While at lower [EY] (< 0.05 mM), there was a direct correlation between [EY] and tensile strength or modulus, at higher [EY] (> 0.05 mM), there was an inverse correlation (Fig. 1a–c & Table S2). The former could originate from incomplete crosslinking of the polymeric network because of lack of EY, and consequently lack of radical initiator^37^. However, the latter could stem from the presence of too many radical species in the reaction media in close proximity, leading to recombination or disproportionation of those radicals and consequently to the termination^44,45^. These data suggest that there is an optimal concentration range of EY (0.05 mM) to afford the highest tensile modulus and strength. The compression and lap shear measurements offer similar results, indicating that under optimal [EY], the compression modulus and shear strength of hydrogel can be improved up to 160 kPa and 560 kPa, respectively only after 1 min of crosslinking using a white LED light with the intensity of 20 mW/cm^2^ (Fig. 1d–e).

To study the stability of the synthesized hydrogels, which is one of the key components to define its biomedical applications, we used the enzymatic degradation assay. We incubated the hydrogels in a solution containing collagenase and compared the dried weight of the samples to the initial dried weight as a function of exposure time^7^. Consistent with the mechanical properties, hydrogels formed under the optimal [EY] (*ca.* = 0.05 mM) had longer retentions (Fig. 1f & Table S3). The enhanced stability against enzymatic degradation indicates an improved crosslinking density, which in turn can restrict the accessibility of the enzyme to the cleavage sites of the hydrogel while increasing the anchoring points of the network^46^.

The swelling ratio as a function of time shows a minima at (EY = 0.05 mM) (Fig. 1g, h & Table S4). This behavior is consistent with the prior mechanical evaluations and is likely originated from varying crosslinking degrees. We then studied the cytotoxicity of crosslinking conditions on corneal fibroblasts in 3-D cell culture. Quantification of cellular viability, determined by the Live-Dead study, showed an inverse correlation between cell viability and [EY] (Fig. 1i, j). In the lower [EY] (< 0.05 mM) the difference in the cellular viability was not substantial. However, as [EY] was increased, its impact on the viability of the cells became more pronounced.

### Tuning TEOA concentration

**Figure 2:**
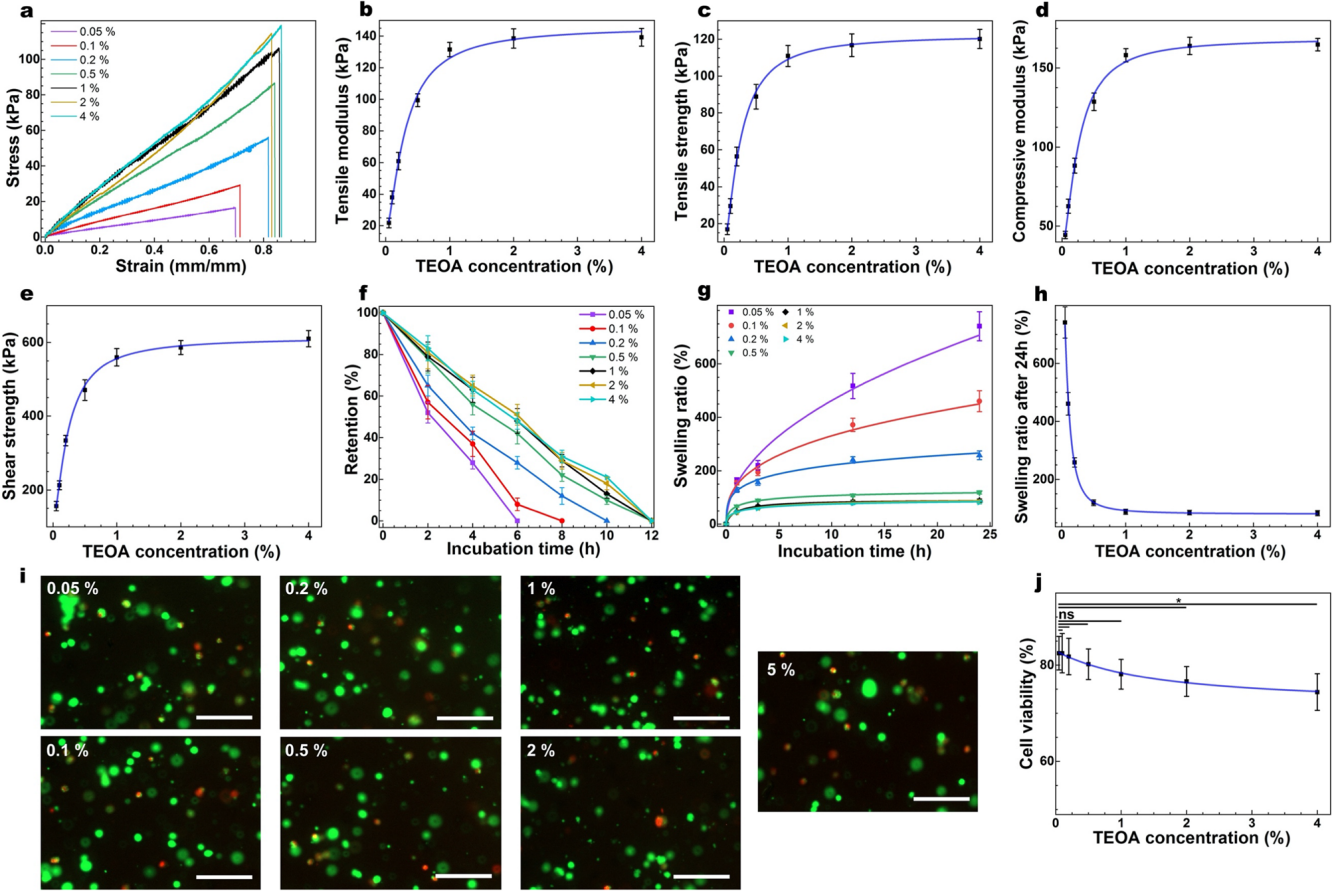
Tuning [TEOA] and its impact on the structural and biological properties of GelMA.(**a**) Representative tensile stress/strain plots for GelMA (20% w/v and crosslinked for 1 min) with varying [TEOA] and their corresponding average tensile modulus (**b**) and tensile strength (**c**), along with mean compressive modulus (**d**), and shear strength (**e**) as a function of [TEOA]. Collagenase induced degradation (**f**) and swelling ratio (**g**) of the GelMA hydrogels formed with varying [TEOA] (20% w/v) as a function of time and corresponding swelling ratios after 24 h of incubation at 37° C. Representative Live-Dead images of the encapsulated CF in 3-D cell culture (**i**) and the quantification of cell viability (**j**) after 1 day of post-seeding on GelMA hydrogels (10% w/v and crosslinked for 1 min) formed with varying [TEOA] (scale bars: 150 μm). Values are presented as mean ± SD; n = 4. ns and *, represent *p* > 0.05 and *p* ≤ 0.05, respectively.

After determining the optimum concentration range of EY ([EY] = 0.05 mM), which afforded the highest tensile modulus and strength, we retained that concentration and tuned [photoinitiator (TEOA)] from 0.05 to 4% (w/v) (0.05, 0.1, 0.2, 0.5, 1, 2, 4%) and studied mechanical, structural, and degradation properties of the hydrogels along with the cytotoxicity of the reaction (Table S1). As shown in Fig. 2a–c, there was a sigmoidal correlation between tensile strength/modulus and [TEOA], ranging from 17 to 120 kPa, and 22–140 kPa, respectively (Table S2). At lower [TEOA] (0.05–0.5% (w/v)), the tensile properties were strongly correlated with [TEOA], indicating lack of initiator at lower [TEOA], slower polymerization kinetics, and incomplete crosslinking at a given time^45^. However, such dependence plateaued at the higher [TEOA] (> 1% (w/v)), suggesting that excess TEOA may not be converted to radical form and consequently may not contribute to the crosslinking reaction as the excited EY limits the radical form of TEOA^44^. The compression modulus and shear strength as a function of [TEOA] showed similar trends, ranging from 44 to 164 kPa and 156–610 kPa, respectively (Fig. 2d, e). The retention of the hydrogel in the collagenase solution was also directly correlated with [TEOA], with the fastest degradation in the hydrogels formed with [TOEA] = 0.05% (6 h) and slowest for the hydrogels formed with [TOEA] > 1% (Fig. 2f & Table S3). The swelling ratio, in contrast, exhibited an inverse sigmoidal correlation with [TEOA] ranging from 83 to 741% after 24 h of incubation in the PBS at room temperature (Fig. 2g, h & Table S4). At lower [TEOA] = 0.05–1% (w/v), the correlation between swelling ratio and [TEOA] was strong, and then plateaued at higher [TEOA] > 1% (w/v). These data agree with mechanical properties assessment and are believed to originate from varying crosslinking degrees between polymeric chains^47,48^.

Cytotoxicity studies also showed an inverse sigmoidal correlation between cell viability and [TEOA] (Fig. 2i, j), demonstrating that for lower [TEOA] (< 0.05 mM), cellular viability was high, yet as [TEOA] was increased, the cellular viability slowly dropped. Such decline viability indicates the toxicity of TEOA at higher concentrations as previously shown^28,49^.

### Tuning VC concentration

**Figure 3:**
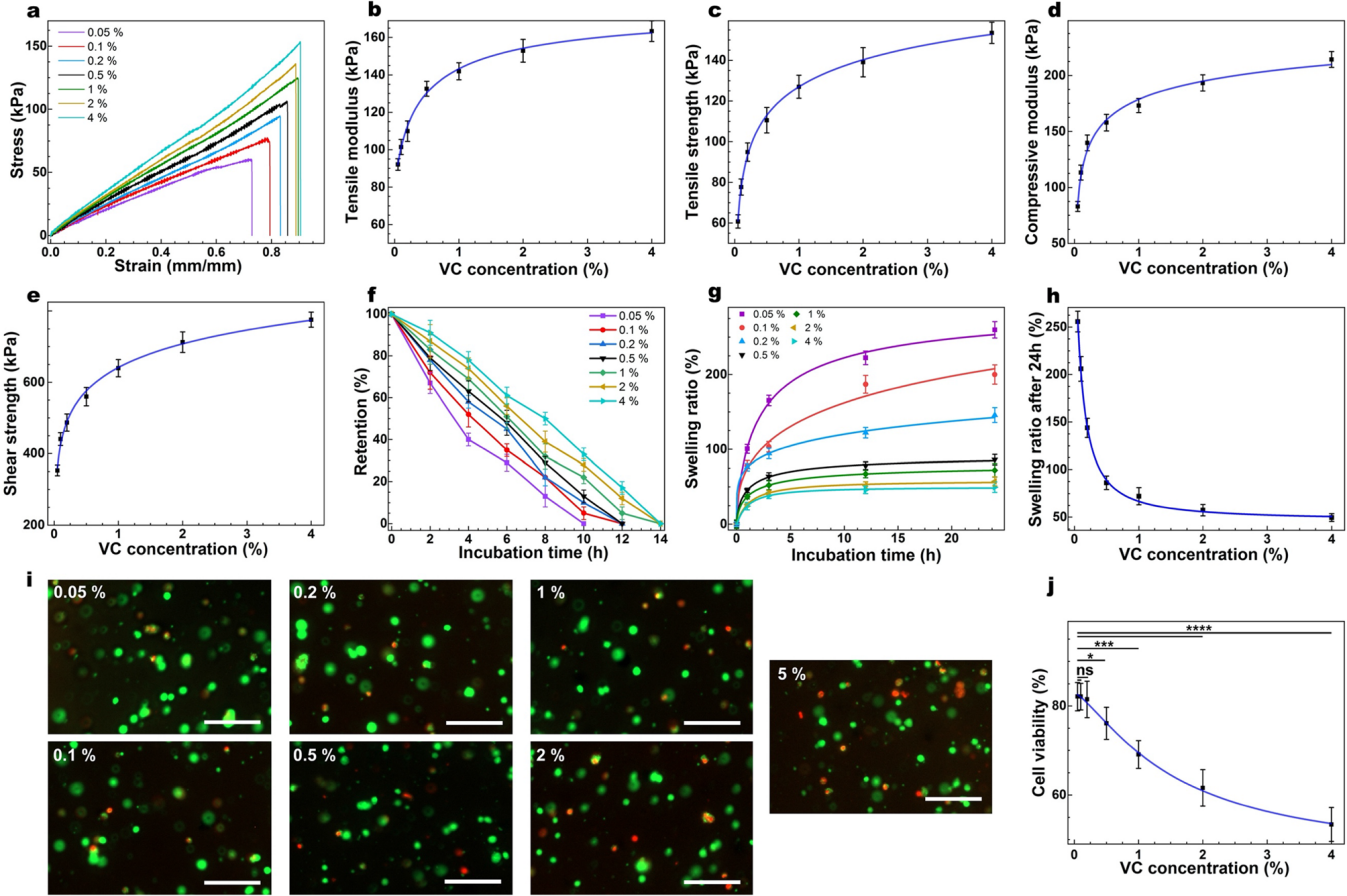
Tuning [VC] and its impact on the structural and biological properties of GelMA. (**a**) Representative tensile stress/strain plots for GelMA (20% w/v and crosslinked for 1 min) with varying [VC] and their corresponding average tensile modulus (**b**) and tensile strength (**c**), along with mean compressive modulus (**d**), and shear strength (**e**) as a function of [VC]. Collagenase induced degradation (**f**) and swelling ratio (**g**) of the GelMA hydrogels formed with varying [VC] (20% w/v) as a function of time, and corresponding swelling ratios after 24 h of incubation at 37 °C. Representative Live-Dead images of the encapsulated CF in 3-D cell culture (**i**) and the quantification of cell viability (**j**) after 1 day of post-seeding on GelMA hydrogels (10% w/v and crosslinked for 1 min) formed with varying [VC] (scale bars: 150 μm). Values are presented as mean ± SD; n = 4. ns, *, **, ***, and **** represent *p* > 0.05, *p* ≤ 0.05, *p* ≤ 0.01, *p* ≤ 0.001 and *p* ≤ 0.0001, respectively.

VC functions as a co-monomer to accelerate the reaction rate and increase the crosslinking density. Without VC the crosslinking can happen, yet with a much slower rate and affords a hydrogel with inferior mechanical properties. Therefore, VC presence is not as vital as EY and TEOA. After finding the optimal [EY] (0.05 mM) and [TEOA] (1% (w/v) highest efficiency before the presence of a plateau), we maintained the concentrations of those crosslinking agents and examined the mechanical, structural, and degradation properties of the hydrogel and their cytotoxicity (Table S1) as a function of [co-monomer (VC)] (0.05, 0.1, 0.2, 0.5, 1, 2, 4%). As illustrated in Fig. 3a–c and Table S2, there is a sigmoidal correlation between tensile strength/modulus and [VC]. Tensile strength/modulus ranged from 60–153 kPa, and 92–164 kPa, respectively. At lower [VC], the tensile properties appeared to correlate strongly with [VC], suggesting the importance of the co-monomer in the crosslinking reaction. Such importance is likely because the addition of co-monomer increases the vinyl group abundance in the reaction, which can synergistically cross‐propagate with methacrylate groups of GelMA while enhancing the diffusion of radicalized species^27,35^ and facilitating free-radical polymerization via preventing the scavenging free-radicals by oxygen^12,36^. On the other hand, at the higher [VC] (> 1% (w/v)) the correlation between tensile properties and [VC] was comparatively less pronounced. This correlation pattern suggests that excess [VC] in the reaction mixture may function only as a co-monomer to cross‐propagate with methacrylate groups of GelMA and thus increase the polymerization kinetics^44,50^. The compression and lap modulus as a function of [VC] showed similar trends, with those ranging from 102–214 kPa and 353–715 kPa, respectively (Fig. 3d, e & Table S2). Therefore, [VC] can be a valuable tool to further enhance the mechanical properties of the desired hydrogel.

Degradation studies showed that the retention of the hydrogel in the collagenase solution was directly correlated with [VC], with the fastest degradation occurring in the hydrogels formed with [VC] = 0.05% (*ca.* 10 h), and slowest degradation in those formed with [VC] > 1% (Fig. 3f & Table S3). The swelling test showed an inverse sigmoidal correlation between the swelling ratio and [VC] (Fig. 3g, h), spanning from 50 to 255% after 24 h of incubation in PBS. This data agrees with the mechanical properties, indicating lower swelling for the hydrogels with superior mechanical properties ([VC] > 1% (w/v)), and higher for the hydrogels with inferior properties ([VC] < 0.5% (w/v)) (Table S4). Higher [VC], which can co-crosslink with GelMA, can decrease the hydrophilicity of the hydrogel, which may contribute to the declined swelling ratio of the hydrogel^51^. The viability of corneal fibroblasts showed an inverse sigmoidal correlation with [VC], suggesting the cytotoxicity of VC at higher concentrations (Fig. 3i, j) as previously shown^28,52^.

### Tuning GelMA concentration

**Figure 4:**
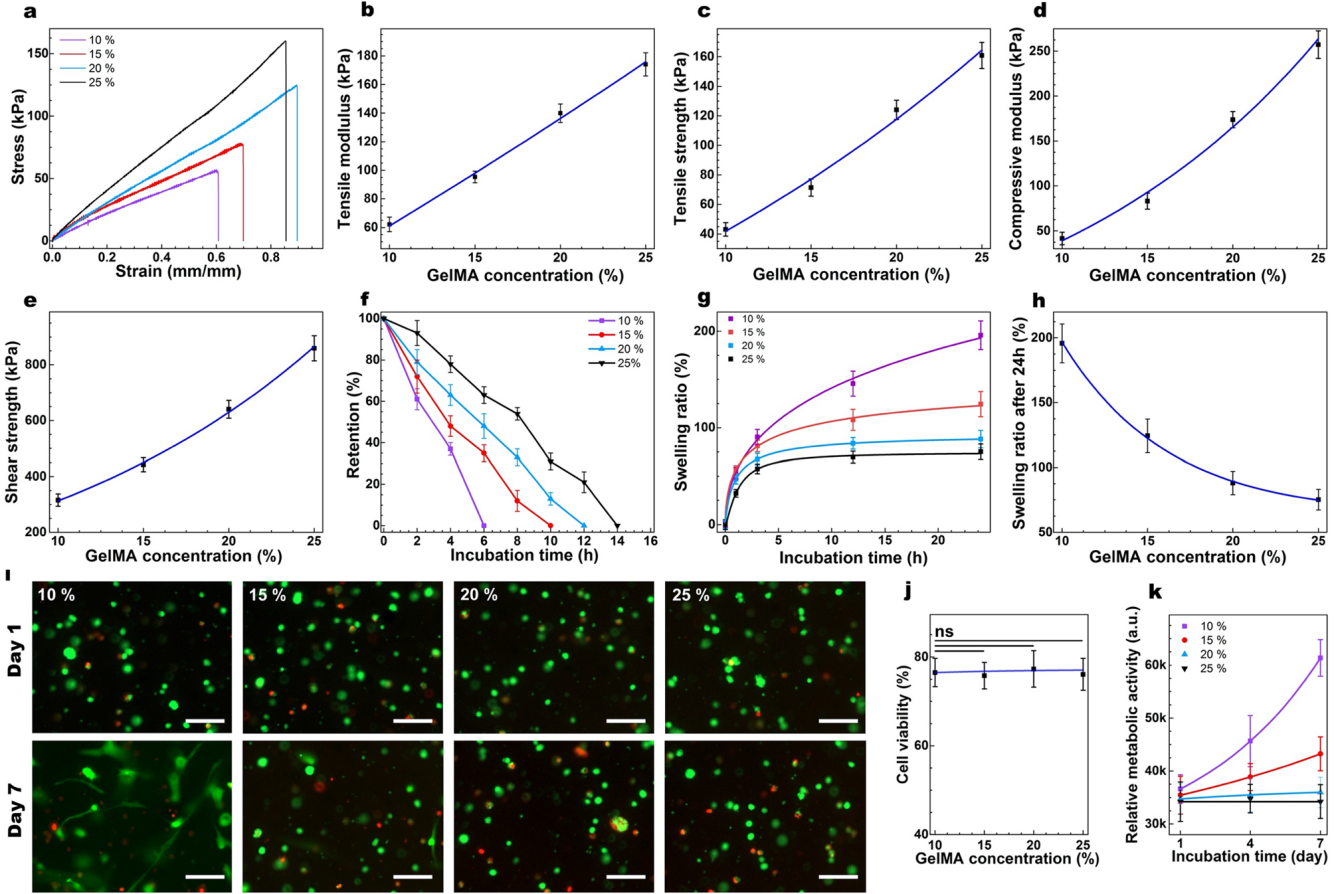
Tuning [GelMA precursor] and its impact on the structural and biological properties of GelMA. (**a**) Representative tensile stress/strain plots for GelMA (crosslinked for 1 min) with varying concentrations and their corresponding average tensile modulus (**b**) and tensile strength (**c**), along with mean compressive modulus (**d**), and shear strength (**e**) as a function of [GelMA precursor]. Collagenase-induced degradation (**f**) and swelling ratio (**g**) of the GelMA hydrogels formed with varying concentrations as a function of time, and corresponding swelling ratios after 24 h of incubation at 37 °C. Representative Live-Dead images of the encapsulated CF in 3-D cell culture (**i**) and the quantification of cell viability (**j**) after 1 and 7 days of post-seeding (crosslinked for 1 min) formed with varying [GelMA] (scale bars: 150 μm). **k**) Quantification of metabolic activity of encapsulated CF on GelMA hydrogels formed with varying [GelMA] after 1, 4, and 7 days of post-seeding. Values are presented as mean ± SD; n = 4. ns represents *p* > 0.05.

After finding the optimal [EY] (0.05 mM) and [TEOA] (1% (w/v)) and [VC] (1% (w/v) highest efficiency before the presence of a plateau), we next retained those concentrations and varied the concentration of GelMA precursor from 10 to 25% (w/v) (10, 15, 20, 25%), and studied how physicochemical properties of the hydrogel behaved under such optimized conditions (Table S1). Tensile strength/modulus as a function of [GelMA precursor] exhibited exponential growth correlations (Fig. 4a–c), with the tensile strength/modulus ranging from 57–161 kPa, and 62–181 kPa, respectively (Table S2). The higher the concentration of GelMA precursor, the higher the abundance of the vinyl-bearing precursor in the close vicinity, and the higher the chance for the propagation of generated radicals as opposed to termination to facilitate the gelation kinetics. Moreover, the hydrogels formed with a higher concentration of GelMA precursor are expected to have higher densities of the polymeric chains with lower porosities, and consequently superior mechanical properties. These explain the exponential dependence of the mechanical properties to [GelMA precursor] and suggest that through increasing precursor concentration, those properties can be significantly improved. The compression modulus and lap shear strength as a function of [GelMA precursor] exhibited similar trends and ranged from 93–245 kPa and 315–859 kPa, respectively (Fig. 4d, e & Table S2).

Enzymatic degradation testing showed the fastest degradation of the hydrogels formed with lower [GelMA precursor] = 10% (w/v) with retention of 4 h) and slowest degradation in the hydrogels formed with higher [GelMA precursor] = 25% (w/v) (Fig. 4f & Table S3), with the retention of hydrogel in the collagenase solution for up to 14 h. The swelling ratio assay indicated an exponential decay correlation between the swelling ratio and [GelMA precursor] (Fig. 4g, h & Table S4) with a swelling ratio ranging from 75 to 195% after 24 h of incubation. This data is consistent with the mechanical properties and is believed to originate from varying porosity of the crosslinked hydrogel as previously shown^5,26,41^.

Cytotoxicity studies, on the other hand, did not show any correlation between cell viability and [GelMA precursor] (Fig. 4i, j). However, most of the encapsulated cells in the hydrogel formed with 10% (w/v) GelMA precursor elongated, spread, and formed interconnected networks with neighboring cells within 7 days of cell culture. Yet, the spreading was very limited in the hydrogel with 15% precursor as previously shown^54^, with no evidence of cellular spreading in the hydrogels formed with 20 and 25% of GelMA precursor (Fig. 4i). The metabolic activities of corneal fibroblasts as a function of incubation time showed a gradual increase, indicated by the relative fluorescence intensities for the hydrogels formed with 10 and 15% GelMA concentrations, suggesting cellular growth and proliferation over time (Fig. 4k). Yet, the metabolic activities of the fibroblasts encapsulated within the hydrogel with a higher concentration of precursor did not change over time after 7 days of cell culture, indicating that higher [GelMA precursor] can slow cell growth and spreading because of enhanced structural and degradation properties of those hydrogels^54^. Yet, most of the encapsulated cells were viable, suggesting an appropriate level of permeability within the hydrogels network formed with a higher concentration of precursor.

### Varying crosslinking time

**Figure 5:**
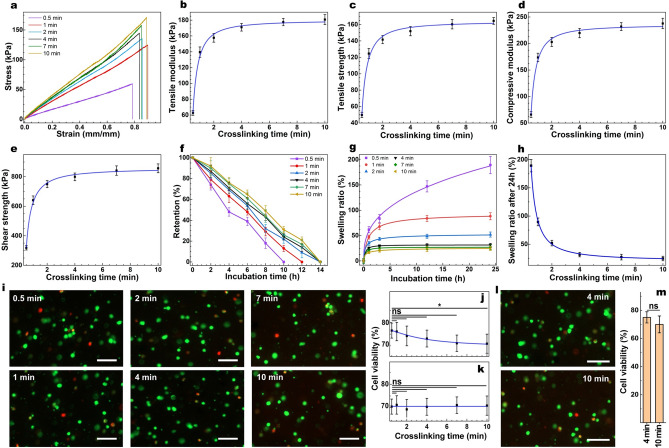
Tuning the crosslinking time and its impact on the structural and biological properties of GelMA. (**a**) Representative tensile stress/strain plots for GelMA (20% w/v) with varying crosslinking time and their corresponding average tensile modulus (**b**) and tensile strength (**c**), along with mean compressive modulus (**d**), and shear strength (**e**) as a function of crosslinking time. Collagenase induced degradation (**f**) and swelling ratio (**g**) of the GelMA hydrogels formed with varying crosslinking time (20% w/v) as a function of time, and corresponding swelling ratios after 24 h of incubation at 37 °C. Representative Live-Dead images of the encapsulated corneal CF in 3-D cell culture (**i**) and the quantification of cell viability after 1 day of post-seeding in GelMA hydrogels (10% w/v) with immediate washing (**j**) and postponed washing (**k**) all after 10 min formed with varying crosslinking time (scale bars: 150 μm). Representative Live-Dead images of the encapsulated mesenchymal stems cells in 3-D cell culture (l) and the quantification of cell viability after 1 day of post-seeding in GelMA hydrogels (20% w/v) crosslinked for 4&10 min. Values are presented as mean ± SD. n = 4; ns and * represent *p* > 0.05 and *p* ≤ 0.05, respectively.

To study the effect of crosslinking time on the physicochemical properties of the hydrogels, we next varied the visible light irradiation time from 0.5 to 10 min, while maintaining the concentrations of crosslinking agents ([EY] = 0.05 mM, [TEOA] = 1% (w/v), [VC] = 1% (w/v), and [GelMA precursor] = 20% (w/v) (Table S1). Tensile assessments indicated a sigmoidal correlation between crosslinking time and tensile modulus/strength (Fig. 5a–c), spanning from 49–181 kPa, and 56–164 kPa, respectively (Table S2). At the beginning of the visible light irradiation, the tensile properties were strongly correlated with the crosslinking time, suggesting the importance of irradiation on the generation of free radicals. However, such correlation gradually plateaued, particularly after 4 min of irradiation, suggesting that when the crosslinking reaction reaches the completion step, further irradiation may not significantly improve the mechanical properties. The compression and lap shear experiments exhibited similar trends with the compression modulus and shear strength spanning from 65–238 kPa and 319–857 kPa, respectively (Fig. 5d, e & Table S2).

Enzymatic degradation testing showed faster degradation of the hydrogels exposed to shorter light durations, and slower degradations for the hydrogels with longer exposures, with the retention time of 10–14 h in the collagenase solution (Fig. 5f & Table S3). The swelling study showed an exponential decay correlation between the swelling ratio, which ranged from 25–189% after 24 h of incubation and crosslinking time (Fig. 5g, h & Table S4). These data are consistent with the structural properties and indicate the importance of varying crosslinking degrees as previously shown^54^. Cytotoxicity studies suggested an inverse correlation between cell viability and crosslinking time (Fig. 5i, j). Such cytotoxicity could originate from prolonged exposure to the crosslinking agents during the gelation reaction. To normalize the effect of exposure to crosslinking agents, we generate a series of hydrogels with varying crosslinking time, yet with the same exposure time to the crosslinking reagents as they all were rinsed after 10 min. Quantification of cellular viability shown by the Live-Dead assay suggested that similar cell viability across all groups (Fig. 5k). This data verifies that prolonged exposure to the crosslinking agents reduces cell viability and indicates the importance of the concentration of crosslinking reagents and exposure time in cell encapsulation studies. To understand how the optimized conditions impact the viability of other types of cells such as human mesenchymal stem cells (MSCs), which are the prominent subject in the modern research era due to their importance in tissue engineering and regenerative medicine^55^, we photoencapsulated them in GelMA solution containing (20% (w/v) GelMA, 0.05 mM EY, 1% (w/v) TEOA, and 1% (w/v) VC. Cytotoxicity studies showed that MSCs have high viability ~ 75% and 72% after 4 and 10 min of light exposure (Fig. 5l, m). While we did not find a statistically significant difference between the viability of cells crosslinked for 4 and 10 min, the general trend of declining viability as a function of exposure is evident. This data suggests that the crosslinking conditions have a similar impact on other types of cells.

Overall, in this report, we performed a systematic study of the impacts of tuning the concentrations of crosslinking reagents, [GelMA precursor], and crosslinking times, generated a library of GelMA hydrogels, and studied their corresponding chemical, physical, mechanical, and biological properties. We determined an optimal set of crosslinking conditions under visible light irradiation, which may permit us to engineer a hydrogel with tailored properties essential for specific biomedical applications.

## Methods

### Materials

All chemicals were used as received from Sigma-Aldrich (St. Louis, MO) without further purification unless otherwise mentioned. No animals or human participant was involved in the study, and only the fibroblast cell line (kind gift of Dr. J. Jester, UC-Irvine) was used to perform biocompatibility studies.

### Chemical synthesis of GelMA

To synthesize GelMA with a high functionalization degree, gelatin (10 g, 300 g Bloom, type A) was dissolved in 100 mL PBS solution, followed by the addition of 8 mL methacrylate anhydride as previously described^22^. The reaction mixtures were stirred for 3 h at 50 °C, diluted with 100 mL deionized (DI) water, and dialyzed for 7 days using a dialysis membrane with a molecular weight cut-off of 14 kDa (Sigma-Aldrich). Next, the GelMA solution was freeze-dried for 5 days to obtain a foam-like GelMA precursor.

### Preparation of GelMA pre-polymer solutions

*Varying [EY]*. To prepare GelMA solutions with various [EY], 1.00 g GelMA was dissolved in 3.1 mL PBS at 45 °C. Then, the solution was mixed with 200 μL of 25% (w/v) TEOA, 500 μL of 5.0% (w/v) VC, and varying contents of EY (1.0, 2.0, 4.0, 10, 20, 40, 100, and 200 μL of EY with the concentration of 25 mM (0.173 g/10 mL)) in PBS in the dark, and addition of PBS to make 5.0 mL GelMA solution, and agitated at 45 °C.

*Varying [TEOA]*. To prepare GelMA solutions with varying [TEOA], 1.00 g GelMA was dissolved in 2.6 mL PBS at 45 °C. Then, the solution was mixed with 500 μL of 5.0% (w/v) VC, 10 μL of EY (25 mM), and varying TEOA contents (10, 20, 40, 100, 200, 400, 800 μL of TEOA (25% w/v) in PBS in the dark, followed by addition of extra PBS to make 5.0 mL GelMA solution, and agitated at 45 °C.

*Varying [VC]*. To prepare GelMA solutions with varying [VC], 1.00 g GelMA was dissolved in 2.6 mL PBS solution at 45 °C. Then, the solution was mixed with 10 μL of EY (25 mM), of 200 μL of TEOA (25% w/v), and varying VC contents (50, 100, 200, and 500 μL of VC (5% w/v), 0.05, 0.1, and 0.2 g of VC) in PBS solution in the dark, followed by addition of extra PBS to make 5.0 mL GelMA solution, and agitation at 45 °C.

*Varying [GelMA]*. To prepare GelMA with varying concentrations, different amount of GelMA (0.5, 0.75, 1, and 1.25 g) was dissolved in 3.1 mL PBS at 45 °C. Then, the solution was mixed with 10 μL of EY (25 mM), of 200 μL of TEOA (25% w/v), and 0.1 g of VC in PBS in the dark conditions, followed by the addition of extra PBS to make 5.0 mL GelMA solution, and agitation at 45 °C.

### Crosslinking conditions

After preparing the reaction mixtures as described above, the prepared solutions were carefully transferred to an appropriate mold and crosslinked for varying times (0.5, 1, 2, 4, 7, and 10 min) using our hand-made visible light source (LED with a wavelength of 490–510 nm and the intensity of 20 mW/cm^2^).

### Mechanical characterization

Mechanical properties studies were conducted using a mechanical tester (Instron mechanical tester; Norwood, MA) as previously described^4,56^. For *tensile* tests, dumbbell-shaped hydrogels were prepared and stretched at a rate of 2 mm/min until rupture, and the stress was recorded as a function of the strain. The tensile modulus was calculated from linear derivatives of the stress–strain plots at 0–60% strain [n = 4]. For the *compression* tests, disk-shaped samples were used and compressed with a crosshead speed of 0.5 mm/min until the maximum stress of 0.6 MPa^4,57^. The compressive stress was recorded as a function of the strain. The compressive modulus for each sample was determined from the linear derivatives of the stress–strain curves at 0–10% strain [n = 4]. *Lap Shear* strength of GelMA hydrogels was measured using a modified lap shear test according to ASTM F2255-05 standard lap shear test. First, a glass slide (10 × 40 mm diameter) was functionalized with 3-(trimethoxysilyl)propyl methacrylate as previously described^58^. Then, 20 μL of GelMA precursor solution was transferred on one slide and the other slide was carefully placed on the GelMA solution, and photo-crosslinked by visible light. The shear strengths of the samples were then determined using a mechanical tester with a crosshead speed of 1 mm/min, and the shear strengths of the GelMA were recorded at the detachment point [n = 4].

### In vitro biodegradation

Enzymatic degradation of GelMA hydrogels was determined using collagenase from *Clostridium histolyticum*, as previously reported^7^. Briefly, we first generated disc-shape constructs, rinsed them with water, and placed them in a solution containing collagenase (40 μg/mL) in Tris-HCl buffer (0.1 M, pH of 7.4), supplemented with CaCl_2_ (5 mM) and incubated at 37 °C. The collagenase solution was changed every 8 h and the hydrogel residue was carefully removed from the solution, rinsed with water, lyophilized, and then its dried mass at different time points (*W*_*f*_) was measured. W_i_ is the weight of discs after lyophilization. The degradation rate was calculated [n = 4] using the following equation:$Residual mass\left(\%\right)= \frac{{W}_{f}}{{W}_{i}} \times 100$

### Swelling ratio

To determine the swelling ratio, first, we generated disc-shape constructs, rinsed them with water, and blot dried them to obtain their initial wet weights (*W*_*i*_). Those discs were then immersed in PBS solution and incubated at 37 °C for up to 24 h. After predetermined periods (2, 4, 12, 24 h), the swollen hydrogel samples were blot dried, and their swollen weights (*W*_*s*_) were measured. The swelling ratio (*S*) for the hydrogels [n = 4] was calculated according to the following equation and plotted as a function of time. $S\left(\%\right)=\frac{\left({W}_{s}-{W}_{i}\right)}{{W}_{i}} \times 100$

### In vitro and ex vivo biocompatibility

*Cell encapsulation (3D culture)*. Fibroblast cells were trypsinized and resuspended (2 × 10^5^ cells per mL) in the hydrogel precursor (10% GelMA solution with varying crosslinking concentrations as described above). Then, 20 μL of the cell dispersion was transferred to polydimethylsiloxane (PDMS) mold (diameter = 8 mm, thickness = 0.2 mm) and exposed to photoirradiation for a varying period. Those cell-laden gels were immediately washed 3 times with PBS and incubated in 200 μL culture medium (DMEM/F-12 50/50, 1 × media (Corning, VA, USA) at 37°C^59^. For mesenchymal stems cells, after resuspending (2 × 10^5^ cells per mL) in the hydrogel precursor solution containing (10% GelMA, 0.05 mM EY, 1% (w/v) TEOA, and 1% (w/v), the suspension (20 μL) was transferred to PDMS mold and exposed to photoirradiation for a 4 and 10 min. Those cell-laden gels were immediately washed 3 times with PBS and incubated in 200 μL of an optimized serum-containing medium (catalog # 02,100, Reachbio lab, Spokane, WA) at 37 °C.

*Cell viability.* The viability of cells was assessed using a commercial kit (LIVE/DEAD™ viability/cytotoxicity kit, for mammalian cells, (Thermofisher Scientific; Cambridge, MA), as previously described^60,61^. Briefly, cells were double-stained with calcein acetoxymethyl and ethidium homodimer for 30 min and imaged by inverted fluorescent microscopy (Zeiss Axio Observer Z1; Thornwood, NY) with a 10X objective. The number of live and dead cells was quantified using the ImageJ software (NIH, Bethesda, Maryland), and the cell viability was assessed from the number of live cells divided by the total number of live and dead cells^60^. Three samples were used for each condition and four images were acquired from each sample.

*AlamarBlue Assay.* To assess the metabolic activity of the encapsulated cells in GelMA, we used a standard AlamarBlue assay as explained elsewhere^62^. Briefly, cell-laden gels were incubated at 37 °C and 5% CO_2_ conditions for up to 7 days. The AlamarBlue test was performed on days 1, 4, and 7 after cell seeding. At each time point, the tissue culture media was replaced with fresh media (200 μL) containing resazurin sodium salt (0.004% w/v) and incubated for 2 h. Afterward, the media (100 μL) was transferred into a new 96 well plate and read on a BioTek plate reader (Synergy 2, BioTek Instruments; Winooski, VT) at 530/25 nm for excitation and 600/25 nm for emission and corrected with the fluorescence of GelMA discs incubated without cells. Four samples for each group and 3-time points were tested.

### Data analysis

Mechanical properties, swelling ratios, enzymatic degradation, and cytotoxicity data as a function of reaction conditions were plotted using OriginLab 2018, and the data fitting process was performed using exponential growth and decay and sigmoidal functions as described in the main text.

### Statistical analysis

One-way ANOVA with Tukey comparison test on GraphPad Prism Software (GraphPad Software version 9.2.0, CA, USA) was used to compare cell viability between groups. A value of *p* ≤ 0.05 was considered statistically significant. n.s., *, **, ***, and **** represent *p* > 0.05, *p* ≤ 0.05, *p* ≤ 0.01, *p* ≤ 0.001 and *p* ≤ 0.0001, respectively.

## Supplementary Information

Supplementary Information.
