# Supplementary material for: Systematic optimization of visible light-induced crosslinking conditions of gelatin methacryloyl (GelMA)
Source: Sci Rep. 2021 Dec 2;11:23276. doi: 10.1038/s41598-021-02830-x (PMC8640009; doi:10.1038/s41598-021-02830-x)
Supplement: Supplementary file 1 — Supplementary Information. [file 41598_2021_2830_MOESM1_ESM.docx]

Supporting Information

**Systematic optimization of visible light-induced crosslinking conditions of gelatin methacryloyl (GelMA)**

**Sina Sharifi, Hannah Sharifi, Ali Akbari, and James Chodosh**

**Table S1**. GelMA precursor formulations

| **Experimental set** | **Entry** | **[EY] (mM)** | **[TEOA] (w/v%)** | **[VC] (w/v%)** | **[GelMA] (w/v%)** | **Crosslinking time (min)** |
| --- | --- | --- | --- | --- | --- | --- |
| Varying [EY] | E1 | 0.005 | 1 | 0.5 | 20 | 1 |
|  | E2 | 0.01 | 1 | 0.5 | 20 | 1 |
|  | E3 | 0.02 | 1 | 0.5 | 20 | 1 |
|  | E4 | 0.05 | 1 | 0.5 | 20 | 1 |
|  | E5 | 0.1 | 1 | 0.5 | 20 | 1 |
|  | E6 | 0.2 | 1 | 0.5 | 20 | 1 |
|  | E7 | 0.5 | 1 | 0.5 | 20 | 1 |
|  | E8 | 1 | 1 | 0.5 | 20 | 1 |
| Varying [TEOA] | T1 | 0.05 | 0.05 | 0.5 | 20 | 1 |
|  | T2 | 0.05 | 0.1 | 0.5 | 20 | 1 |
|  | T3 | 0.05 | 0.2 | 0.5 | 20 | 1 |
|  | T4 | 0.05 | 0.5 | 0.5 | 20 | 1 |
|  | T5 | 0.05 | 1 | 0.5 | 20 | 1 |
|  | T6 | 0.05 | 2 | 0.5 | 20 | 1 |
|  | T7 | 0.05 | 4 | 0.5 | 20 | 1 |
| Varying [VC] | V1 | 0.05 | 1 | 0.05 | 20 | 1 |
|  | V2 | 0.05 | 1 | 0.1 | 20 | 1 |
|  | V3 | 0.05 | 1 | 0.2 | 20 | 1 |
|  | V4 | 0.05 | 1 | 0.5 | 20 | 1 |
|  | V5 | 0.05 | 1 | 1 | 20 | 1 |
|  | V6 | 0.05 | 1 | 2 | 20 | 1 |
|  | V7 | 0.05 | 1 | 4 | 20 | 1 |
| Varying [GelMA] | G1 | 0.05 | 1 | 1 | 10 | 1 |
|  | G2 | 0.05 | 1 | 1 | 15 | 1 |
|  | G3 | 0.05 | 1 | 1 | 20 | 1 |
|  | G5 | 0.05 | 1 | 1 | 25 | 1 |
| Varying crosslinking time | t1 | 0.05 | 1 | 1 | 20 | 0.5 |
|  | t2 | 0.05 | 1 | 1 | 20 | 1 |
|  | t3 | 0.05 | 1 | 1 | 20 | 2 |
|  | t4 | 0.05 | 1 | 1 | 20 | 4 |
|  | t5 | 0.05 | 1 | 1 | 20 | 7 |
|  | t6 | 0.05 | 1 | 1 | 20 | 10 |

**Table S2.** Mechanical properties of the hydrogels

| **Entry** | **Tensile Modulus (kPa)** | **Tensile Strength (kPa)** | **Compression Modulus (kPa)** | **Shear Strength (kPa)** |
| --- | --- | --- | --- | --- |
| E1 | 78.7 ± 3.1 | 59.9 ± 5.1 | 105.6 ± 3.4 | 404.4 ± 14.4 |
| E2 | 97.2 ± 4.2 | 77.2 ± 6.0 | 133.3 ± 4.5 | 466.6 ± 15.2 |
| E3 | 118.3 ± 5.5 | 96.4 ± 6.9 | 147.9 ± 5.7 | 527.2 ± 20.5 |
| E4 | 132.5 ± 4.1 | 111.9 ± 7.1 | 159.6 ± 4.6 | 553.7 ± 22.1 |
| E5 | 121.9 ± 5.3 | 104.9 ± 6.2 | 148.9 ± 4.1 | 536.2 ± 18.5 |
| E6 | 102.6 ± 6.1 | 87.5 ± 8.4 | 136.0 ± 4.5 | 481.2 ± 21.1 |
| E7 | 65.8 ± 4.5 | 67.0 ± 4.3 | 113.1 ± 5.2 | 391.0 ± 15.7 |
| E8 | 43.6 ± 3.0 | 53.1 ± 3.5 | 91.8 ± 4.1 | 316.4 ± 14.4 |
| T1 | 21.8 ± 3.2 | 16.9 ± 2.9 | 44.4 ± 2.4 | 156.2 ± 12.4 |
| T2 | 38.0 ± 4.1 | 29.5 ± 4.0 | 62.6 ± 4.5 | 213.0 ± 11.8 |
| T3 | 60.9 ± 4.9 | 56.4 ± 5.1 | 88.2 ± 4.7 | 333.7 ± 14.3 |
| T4 | 99.4 ± 4.5 | 88.8 ± 6.7 | 128.5 ± 5.6 | 470.5 ± 27.2 |
| T5 | 132.5 ± 4.1 | 111.9 ± 7.1 | 159.6 ± 4.6 | 553.7 ± 22.1 |
| T6 | 138.5 ± 6.1 | 116.7 ± 6.2 | 163.9 ± 5.4 | 586.4 ± 18.9 |
| T7 | 139.2 ± 5.5 | 120.1 ± 5.6 | 164.8 ± 4.0 | 610.1 ± 21.3 |
| V1 | 92.1 ± 4.1 | 60.8 ± 3.3 | 82.9 ± 4.4 | 353.5 ± 14.4 |
| V2 | 101.4 ± 4.1 | 77.7 ± 4.1 | 113.2 ± 6.7 | 440.6 ± 17.6 |
| V3 | 109.9 ± 4.1 | 94.9 ± 4.6 | 139.9 ± 7.0 | 487.0 ± 23.8 |
| V4 | 132.5 ± 4.1 | 111.9 ± 7.1 | 159.6 ± 4.6 | 553.7 ± 22.1 |
| V5 | 141.9 ± 4.1 | 127.0 ± 5.7 | 173.0 ± 6.3 | 639.6 ± 23.4 |
| V6 | 152.8 ± 4.1 | 139.1 ± 7.2 | 193.3 ± 7.2 | 712.4 ± 28.8 |
| V7 | 163.4 ± 4.1 | 153.5 ± 5.3 | 214.2 ± 6.9 | 775.9 ± 19.5 |
| G1 | 62.2 ± 5.1 | 43.2 ± 4.5 | 41.6 ± 7.0 | 315.8 ± 21.3 |
| G2 | 95.3 ± 4.1 | 71.4 ± 5.9 | 83.1 ± 8.6 | 442.1 ± 24.5 |
| G3 | 139.9 ± 6.5 | 124.0 ± 6.5 | 173.8 ± 8.9 | 640.1 ± 28.8 |
| G5 | 185.1 ± 8.1 | 160.9 ± 8.9 | 257.3 ± 15.2 | 859.5 ± 34.1 |
| t1 | 62.8 ± 3.1 | 49.8± 3.3 | 65.8 ± 5.5 | 318.9 ± 15.8 |
| t2 | 139.9 ± 6.5 | 124.0 ± 6.5 | 173.8 ± 8.9 | 640.1 ± 28.8 |
| t3 | 157.6 ± 5.5 | 141.6± 4.9 | 202.7 ± 8.4 | 749.9 ± 24.1 |
| t4 | 171.0 ± 4.8 | 151.8± 5.6 | 219.6 ± 8.8 | 799.2 ± 26.3 |
| t5 | 177.6 ± 4.6 | 160.3± 5.7 | 231.9 ± 7.5 | 841.6 ± 31.4 |
| t6 | 180.7 ± 6.2 | 164.4± 4.1 | 237.8 ± 9.9 | 857.1 ± 29.5 |

**Table S3.** Retention of the hydrogels in the collagenase solution

| **Time (h)**  **Entry** | **0** | **2** | **4** | **6** | **8** | **10** | **12** | **16** |
| --- | --- | --- | --- | --- | --- | --- | --- | --- |
| E1 | 100 | 60 ± 5 | 48 ± 3 | 20 ± 3 | 9 ± 2 | 0 | - | - |
| E2 | 100 | 63 ± 8 | 47 ± 6 | 28 ± 3 | 11 ± 3 | 0 | - | - |
| E3 | 100 | 65 ± 5 | 52 ± 3 | 38 ± 3 | 20 ± 4 | 5 ± 4 | 0 | - |
| E4 | 100 | 79 ± 7 | 63 ± 5 | 48 ± 5 | 29 ± 3 | 13 ± 2 | 0 | - |
| E5 | 100 | 81 ± 7 | 65 ± 6 | 52 ± 6 | 30 ± 3 | 12 ± 2 | 0 | - |
| E6 | 100 | 77 ± 8 | 63 ± 5 | 50 ± 4 | 25 ± 4 | 10 ± 3 | 0 | - |
| E7 | 100 | 71 ± 6 | 49 ± 4 | 32 ± 4 | 12 ± 3 | 0 | - | - |
| E8 | 100 | 60 ± 5 | 45 ±5 | 19 ± 2 | 0 | - | - | - |
| T1 | 100 | 52 ± 5 | 28 ± 3 | 0 | - | - | - | - |
| T2 | 100 | 57 ± 8 | 37 ± 6 | 8 ± 3 | 0 | - | - | - |
| T3 | 100 | 65 ± 5 | 42 ± 3 | 28 ± 3 | 12 ± 4 | 0 | - | - |
| T4 | 100 | 78 ± 7 | 56 ± 5 | 42 ± 5 | 22 ± 3 | 10 ± 2 | 0 | - |
| T5 | 100 | 79 ± 8 | 63 ± 5 | 48 ± 5 | 29 ± 3 | 13 ± 2 | 0 | - |
| T6 | 100 | 81 ± 7 | 65 ± 6 | 51 ± 6 | 29 ± 3 | 18 ± 3 | 0 | - |
| T7 | 100 | 83 ± 6 | 63 ± 4 | 48 ± 4 | 31 ± 3 | 21 ± 2 | 0 | - |
| V1 | 100 | 67 ± 5 | 40 ± 3 | 29 ± 4 | 13 ± 5 | 0 | - | - |
| V2 | 100 | 72 ± 8 | 52 ± 6 | 35 ± 3 | 22 ± 4 | 5 ± 3 | 0 | - |
| V3 | 100 | 78 ± 5 | 58 ± 3 | 45 ± 3 | 22 ± 4 | 10 ± 3 | 0 | - |
| V4 | 100 | 79 ± 8 | 63 ± 5 | 48 ± 5 | 29 ± 3 | 13 ± 2 | 0 | - |
| V5 | 100 | 83 ± 7 | 69 ± 5 | 51 ± 6 | 32 ± 6 | 22 ± 3 | 5 ± 3 | 0 |
| V6 | 100 | 87 ± 8 | 74 ± 5 | 56 ± 4 | 39 ± 5 | 28 ± 3 | 12 ± 3 | 0 |
| V7 | 100 | 91 ± 6 | 78 ± 4 | 61 ± 4 | 50 ± 3 | 33 ± 4 | 17 ± 3 | 0 |
| G1 | 100 | 61 ± 5 | 37 ± 3 | 0 | - | - | - | - |
| G2 | 100 | 72 ± 8 | 48 ± 5 | 35 ± 4 | 12 ± 5 | 0 | - | - |
| G3 | 100 | 81 ± 8 | 65 ± 5 | 51 ± 5 | 33 ± 3 | 15 ± 5 | 0 | - |
| G5 | 100 | 93 ± 6 | 78 ± 4 | 63 ± 4 | 54 ± 3 | 31 ± 4 | 21 ± 5 | 0 |
| t1 | 100 | 74 ± 5 | 48 ± 4 | 39 ± 4 | 18 ± 5 | 0 | - | - |
| t2 | 100 | 81 ± 8 | 65 ± 5 | 51 ± 5 | 33 ± 3 | 15 ± 5 | 0 | - |
| t3 | 100 | 85 ± 5 | 69 ± 3 | 54 ± 3 | 32 ± 4 | 22 ± 4 | 9 ± 5 | 0 |
| t4 | 100 | 87 ± 6 | 71 ± 5 | 56 ± 6 | 43 ± 3 | 24 ± 3 | 14 ± 4 | 0 |
| t5 | 100 | 90 ± 7 | 75 ± 5 | 61 ± 6 | 44 ± 6 | 26 ± 3 | 17 ± 4 | 0 |
| t6 | 100 | 92 ± 8 | 76 ± 5 | 65 ± 4 | 49 ± 5 | 31 ± 3 | 21 ± 3 | 0 |

**Table S4.** Swelling ratios in PBS solution

| **Time (h)**  **Entry** | **0** | **1** | **4** | **12** | **24** |
| --- | --- | --- | --- | --- | --- |
| E1 | 0 | 72 ± 6 | 129 ± 8 | 146 ± 11 | 155 ± 13 |
| E2 | 0 | 65 ± 7 | 122 ± 7 | 131 ± 10 | 137 ± 11 |
| E3 | 0 | 61 ± 6 | 106 ± 8 | 111 ± 8 | 114 ± 11 |
| E4 | 0 | 42 ± 4 | 80 ± 6 | 91 ± 7 | 95 ± 10 |
| E5 | 0 | 42 ± 3 | 79 ± 6 | 89 ± 5 | 93 ± 9 |
| E6 | 0 | 73 ± 5 | 111 ± 6 | 134 ± 8 | 146 ± 10 |
| E7 | 0 | 89 ± 7 | 154 ± 8 | 204 ± 7 | 217 ± 12 |
| E8 | 0 | 113 ± 8 | 217 ± 8 | 267 ± 12 | 286 ± 14 |
| T1 | 0 | 163 ± 10 | 221 ± 18 | 517 ± 47 | 741 ± 54 |
| T2 | 0 | 155 ± 8 | 194 ± 12 | 372 ± 25 | 460 ± 39 |
| T3 | 0 | 128 ± 9 | 157 ± 11 | 24 ± 12 | 258 ± 16 |
| T4 | 0 | 67 ± 3 | 89 ± 5 | 108 ± 5 | 119 ± 6 |
| T5 | 0 | 42 ± 4 | 80 ± 6 | 91 ± 7 | 95 ± 10 |
| T6 | 0 | 46 ± 3 | 62 ± 3 | 83 ± 4 | 85 ± 6 |
| T7 | 0 | 46 ± 3 | 60 ± 4 | 80 ± 6 | 83 ± 6 |
| V1 | 0 | 101 ± 6 | 165 ± 7 | 222 ± 9 | 260 ± 11 |
| V2 | 0 | 78 ± 7 | 103 ± 7 | 187 ± 12 | 200 ± 13 |
| V3 | 0 | 76 ± 5 | 95 ± 7 | 122 ± 6 | 146 ± 10 |
| V4 | 0 | 42 ± 4 | 80 ± 6 | 91 ± 7 | 95 ± 10 |
| V5 | 0 | 37 ± 3 | 52 ± 6 | 67 ± 5 | 72 ± 9 |
| V6 | 0 | 26 ± 2 | 42 ± 3 | 52 ± 5 | 57 ± 6 |
| V7 | 0 | 24 ± 5 | 38 ± 4 | 46 ± 5 | 50 ± 7 |
| G1 | 0 | 53 ± 6 | 90 ± 8 | 146 ± 13 | 196 ± 15 |
| G2 | 0 | 57 ± 4 | 81 ± 6 | 108 ± 11 | 124 ± 13 |
| G3 | 0 | 47 ± 5 | 68 ± 6 | 84 ± 6 | 88 ± 9 |
| G5 | 0 | 32 ± 4 | 58 ± 5 | 70 ± 6 | 75 ± 8 |
| t1 | 0 | 62 ± 6 | 83 ± 8 | 147 ± 11 | 189 ± 17 |
| t2 | 0 | 47 ± 5 | 68 ± 6 | 84 ± 6 | 88 ± 9 |
| t3 | 0 | 35 ± 4 | 43 ± 3 | 49 ± 4 | 52 ± 5 |
| t4 | 0 | 25 ± 2 | 30 ± 2 | 31 ± 3 | 32 ± 3 |
| t5 | 0 | 21 ± 3 | 25 ± 4 | 26 ± 4 | 27 ± 5 |
| t6 | 0 | 17 ± 2 | 20 ± 3 | 23 ± 5 | 25 ± 4 |
